# Dual-Task Performance as a Potential Digital Biomarker of Aging: Age-Stratified Reference Patterns Across the Adult Lifespan

**DOI:** 10.3390/jcm15187234

**Published:** 2026-09-17

**Authors:** Manuel Murie-Fernández, Naiara Mimentza Larrinaga, Marina Cabrera Brito, Carmen Bahamonde Román, Maria Carbo Valle, Soledad Castillo Sarango, Iratxe Arias Izquierdo, Juan Ignacio Marin Ojea, Rafael Valenti Azcarate

**Affiliations:** 1 Neurorehabilitation Unit, Hospital ICOT Ciudad de Telde, 35212 Las Palmas de Gran Canaria, Spain; direcciontecnica@grupoicot.es (M.C.B.); medico2.hct@grupoicot.es (C.B.R.); 2 Neurorehabilitation Unit, Fundación Hospitalarias Euskadi, 20500 Arrasate-Mondragón, Spain; naiara.mimentza@fundacionhospitalarias.org (N.M.L.); iratxe.arias@fundacionhospitalarias.org (I.A.I.); juanignacio.marin@fundacionhospitalarias.org (J.I.M.O.); 3 Neurology Department, Clinica San Miguel, 31006 Pamplona, Spain; rafael.valenti@clinicasanmiguel.es; 4 Medical School, University of Barcelona, 08036 Barcelona, Spain; mariacarbovalle@gmail.com; 5 Facultad de Ciencias de la Salud, Universidad Técnica Particular de Loja, Loja 1101608, Ecuador; smcastillo1@utpl.edu.ec

**Keywords:** dual-task performance, dual-task cost, aging, digital assessment, motor-cognitive interference, neurorehabilitation

## Abstract

**Objectives:** To characterize age-stratified reference patterns in dual-task performance and dual-task cost across the adult lifespan using a standardized digital assessment. **Methods:** A cross-sectional observational study was conducted in 400 healthy participants aged 18 to 94 years from Spain and Ecuador. Participants performed a digital dual-task test combining a motor task (cursor control to hit a virtual target) and a cognitive task (arithmetic operations). Performance was assessed under single-task (ST) and dual-task (DT) conditions. Dual-task cost (DTC) was calculated separately for the motor and cognitive components, together with a composite Mean DTC. Participants were grouped into seven age categories for descriptive age-stratified analyses, and differences were assessed using one-way ANOVA with Bonferroni-adjusted post hoc comparisons. In addition, multiple linear regression analyses were performed with age treated as a continuous variable and sex as a covariate. **Results:** Motor and cognitive performance was lower across older age groups under both ST and DT conditions. DTC differed significantly across age groups (*p* < 0.001), with larger differences becoming apparent at older ages. Cognitive DTC showed a marked increase across older age groups, indicating substantial age-associated cognitive interference under dual-task conditions. Mean DTC increased from 28.1% in participants aged 18–29 years to 63.2% in those aged ≥80 years. Age remained significantly associated with Motor DTC (B = 0.506 per year; 95% CI 0.422–0.589; *p* < 0.001), Cognitive DTC (B = 0.582; 95% CI 0.497–0.667; *p* < 0.001), and Mean DTC (B = 0.544; 95% CI 0.483–0.604; *p* < 0.001) after adjustment for sex. Although adjusted DTC levels differed between sexes, no significant age × sex interaction was observed. **Conclusions:** Digital dual-task performance differed substantially across age groups, with higher dual-task costs observed at older ages. These findings provide age-stratified reference data for this specific digital cognitive-motor paradigm and support its further investigation as a scalable measure of cognitive-motor interference. Further psychometric, longitudinal, and clinical validation is required before the Dualtask Test can be established as a clinical biomarker or used to distinguish physiological age-associated differences from pathological cognitive decline.

## 1. Introduction

Dual-task performance, defined as the simultaneous execution of a motor and a cognitive task, is a complex process that requires efficient coordination of attentional and processing resources [1]. This ability has been widely recognized as a sensitive indicator of functional capacity and overall cognitive status, particularly in aging populations [2].

The integration of cognitive and motor domains during dual-task execution involves significant activation of the prefrontal cortex, reflecting increased cognitive demand compared to single-task conditions [3]. Although older adults may adaptively prioritize one task over another, dual-task cost remains consistently higher compared to younger individuals [4]. Consequently, motor-cognitive dual-task paradigms have become a key area of research for understanding age-related functional decline [2].

A central concept in dual-task research is the dual-task cost (DTC), which quantifies the relative change in performance when two tasks are performed simultaneously compared to when each task is executed individually. DTC is typically calculated as:

DTC (%) = [(Dual-task performance − Single-task performance)/Single-task performance] × 100

This metric provides an estimate of the interference between cognitive and motor domains and allows comparison across individuals and populations [5,6]. Higher DTC values indicate greater performance deterioration under dual-task conditions.

From a neurophysiological perspective, dual-task performance reflects the interaction between motor and cognitive systems, which are closely interdependent. Interference between these domains may lead to measurable changes in performance, commonly quantified through DTC [7]. Aging-related declines in dual-task ability have been linked to structural and functional changes in brain regions such as the cerebellum, basal ganglia, and prefrontal cortex, as well as reduced white matter integrity [4,8,9,10]. These alterations contribute to decreased efficiency in attentional resource allocation and reduced capacity for simultaneous information processing [11].

Furthermore, neuroimaging studies suggest that younger adults exhibit greater prefrontal activation during dual-task performance, whereas older adults show reduced activation and altered neural resource allocation strategies [12]. These findings support the concept of dual-task performance as a marker of neurocognitive aging.

Despite the growing body of research in this field, there is currently no gold standard for the assessment of dual-task performance. A wide variety of paradigms, task combinations, and outcome measures have been used, limiting comparability across studies and hindering the establishment of normative reference values [13]. This heterogeneity underscores the need for standardized and scalable assessment tools.

Recent advances in digital health technologies have enabled the development of objective and reproducible tools for assessing dual-task performance. These systems allow precise quantification of motor and cognitive components and may facilitate large-scale data collection in both clinical and research settings.

In this context, the free online DualtaskTest designed by Dualebike, provides a digital platform designed to quantify dual-task capacity through standardized protocol. However, normative data across a wide age range remain limited.

The aim of this study was to evaluate dual-task performance in a large cohort of healthy individuals across the adult lifespan and to characterize age-related changes in dual-task cost. We hypothesized that dual-task performance would decline progressively with age, reflecting reduced cognitive-motor integration efficiency.

## 2. Materials and Methods

### 2.1. Study Design and Participants

A cross-sectional observational study was conducted in a cohort of 400 healthy participants aged between 18 and 94 years. Participants were recruited in two countries, Spain and Ecuador, using a convenience sampling strategy. Recruitment sources included friends, relatives, and acquaintances of the research team. In the older age groups, additional participants were recruited through the Neurology Department, including accompanying relatives of patients and individuals attending the neurology outpatient clinic who fulfilled the study eligibility criteria. Recruitment through the Neurology Department therefore did not imply the presence of a neurological disorder. All participants were required to meet the same eligibility criteria regarding the absence of known neurological or psychiatric disorders that could affect task performance. In participants aged over 70 years, an additional cognitive screening using the Montreal Cognitive Assessment (MoCA) was performed. Given the higher likelihood of unrecognized cognitive impairment in this age group, this additional screening was implemented as a precautionary measure to reduce the likelihood of including individuals with clinically relevant cognitive impairment. Only participants with scores within the normal range were included. Recruitment therefore followed a convenience sampling strategy rather than a population-based sampling approach.

Inclusion criteria were: (1) age ≥18 years, (2) absence of known neurological, psychiatric, or musculoskeletal disorders that could affect motor or cognitive performance and (3) participants were required not to be taking medications that could impair cognitive or physical performance.

Exclusion criteria included the presence of any condition or treatment that could interfere with task execution or performance.

Educational level was not systematically recorded in this study. Because DTC is calculated within subjects by comparing each participant’s dual-task performance with their own corresponding single-task performance, the within-subject design may reduce some inter-individual variability related to educational background. However, it does not eliminate the potential influence of education on task performance or on comparisons between age groups.

All participants provided informed consent prior to participation.

The study was approved by the Ethics Committee for Research of the Government of Navarra (reference: PI_2024/125) and was conducted in accordance with the Declaration of Helsinki.

### 2.2. Dual-Task Assessment

Dual-task performance was assessed using a standardized digital protocol implemented through the Dualtask Test, developed by M.M.-F. through Dualebike, a company co-founded by M.M.-F. and focused on the development of digital tools for the assessment and training of dual-task performance. The Dualtask Test was specifically developed to provide a scalable and standardized assessment of cognitive-motor dual-task performance.

The test evaluates the ability to perform motor and cognitive tasks both independently and simultaneously and consists of three sequential conditions: (1) motor single-task, (2) cognitive single-task, and (3) dual-task condition.

Both the motor and cognitive components of the dual-task test included six predefined levels of difficulty. In the motor task, difficulty was progressively increased by reducing the size of the ball, thereby requiring greater precision and control. In the cognitive task, difficulty was modulated by varying the complexity and numerical range of arithmetic operations as follows: level 1 consisted of addition tasks within the range 0–10; level 2 included both addition and subtraction within 0–10; level 3 consisted of addition within the range 1–20; level 4 included both addition and subtraction within 0–20; level 5 consisted of addition within the range 0–60; and level 6 included both addition and subtraction within 0–60.

Prior to the present study, preliminary pilot testing was conducted in a separate sample of healthy individuals covering a broad adult age range, with at least 10 participants represented in each decade. The selection of the difficulty level was based on the assessment of task performance by the neurologists and neuropsychologists involved in the development of the test. Levels 1–3 were considered insufficiently demanding, whereas levels 5–6 were considered excessively demanding, particularly because their greater difficulty reduced the number of arithmetic operations that could be completed within the one-minute assessment period, potentially limiting the ability to detect differences in performance. Level 4 was therefore selected as an intermediate level that provided sufficient task demand while remaining feasible across the broad age range included in the study. The same difficulty level (level 4) was used for both the motor and cognitive components of the protocol rather than selecting the difficulty level of each component individually. This preliminary phase was intended to optimize task difficulty and duration and was not designed as a formal psychometric validation study or as a quantitative assessment of floor or ceiling effects.

### 2.3. Test Procedure

Participants were seated comfortably in front of a computer with an external mouse. The Dualtask Test was designed as a web-based assessment that can be administered using a conventional computer. Testing was generally performed using the investigator’s or clinic’s computer and always using a hand-operated computer mouse. Screen size, screen resolution, mouse model, and mouse sensitivity were not standardized across testing sites. However, for each participant, single-task and dual-task conditions were completed during the same session using the same computer, screen, mouse, and hardware configuration. Thus, hardware conditions remained constant within participants for the calculation of DTC. The dominant hand was used for the motor task, while the non-dominant hand was used for the cognitive task.

Before the formal assessment, participants completed an approximately one-minute familiarization phase consisting of 30 s of practice with the motor task followed by 30 s of practice with the cognitive task. These practice periods were not included in the recorded test performance. Following familiarization, the formal assessment comprised three one-minute conditions: one minute of the motor task alone, one minute of the cognitive task alone, and one minute of simultaneous motor and cognitive performance under the dual-task condition.

**Figure 1:**
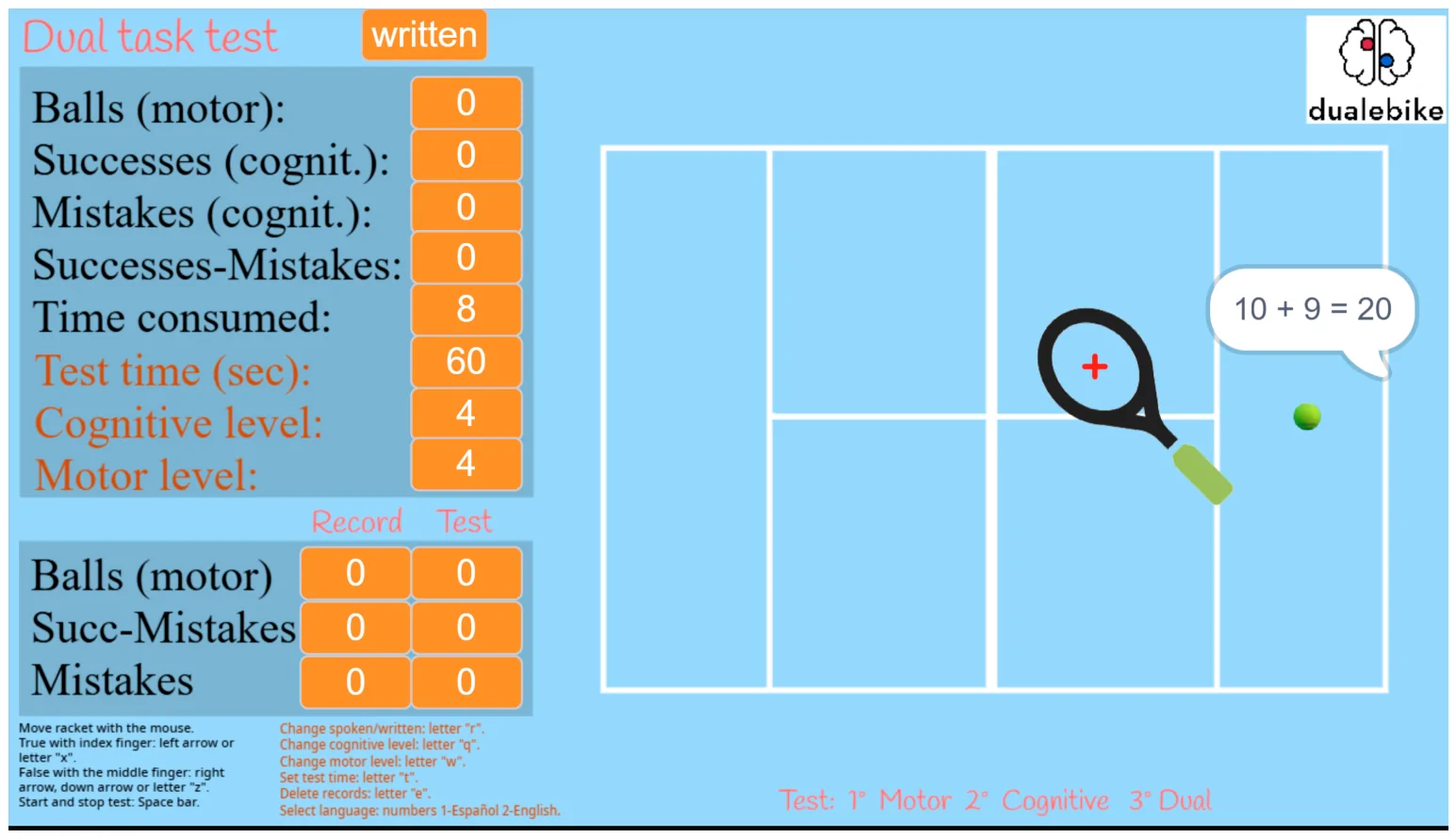
**The Dual-task Test digital assessment.** Representative screenshots of the standardized digital dual-task assessment. Motor single-task: participants control a virtual paddle with the dominant hand using a computer mouse to hit a target. Cognitive single-task: participants solve true/false arithmetic operations using the non-dominant hand. Dual-task condition: simultaneous execution of both motor and cognitive tasks.

The test phase (Figure 1) was performed at a standardized difficulty level and lasted a total of 3 min, divided into:

Baseline condition:

60 s motor task

60 s cognitive task

Dual-task condition:

60 s simultaneous motor and cognitive performance

### 2.4. Motor Task

The motor task consisted of controlling a cursor using a mouse to move a virtual paddle and hit a ball as many times as possible within a fixed time period of one minute. The ball remained stationary until it was struck by the paddle, after which it relocated to a new position. The distance of displacement after each hit was constant, ensuring a standardized motor demand across trials. Participants were therefore required to continuously reorient the cursor and reach the ball repeatedly.

In the motor task, difficulty was progressively increased by reducing the size of the ball, thereby requiring greater precision and control. At Level 4, the diameter of the ball corresponded to approximately 4.2% of the displayed length of the tennis court. Because the test is web-based and may be displayed on monitors of different sizes, the absolute physical dimensions of the ball may vary, whereas its size relative to the tennis court remains constant.

Performance was quantified as the total number of successful hits achieved during the task.

### 2.5. Cognitive Task

The cognitive task consisted of responding to simple arithmetic operations involving additions and subtractions with numbers ranging from 1 to 20. Participants were required to indicate whether the result of each operation was true or false by pressing designated keys with the non-dominant hand (index finger for “true” and middle finger for “false”).

The software generated the arithmetic operations randomly for each trial. However, the level of difficulty was standardized, as the sequence systematically alternated between simpler and more complex operations, as well as between correct and incorrect results. This approach ensured a consistent cognitive load across participants while maintaining variability in task presentation.

Performance was recorded as the number of correct responses, errors, and the net score (correct responses minus errors).

### 2.6. Dual-Task Condition

In the dual-task condition, participants performed both tasks simultaneously, combining motor tracking with cognitive decision-making.

### 2.7. Outcome Measures

Primary and secondary outcome measures were derived from performance in single-task and dual-task conditions.

Motor performance: number of successful hits

Cognitive performance: correct responses, errors, and net score

Dual-task performance: performance under simultaneous conditions

Dual-task cost (DTC) was calculated as the percentage change between single-task and dual-task performance for both motor and cognitive domains.

Additionally, a mean dual-task loss (%) was calculated as a global indicator of cognitive-motor interference, defined as the arithmetic mean of motor and cognitive dual-task cost values.

### 2.8. Data Collection

All measurements were automatically recorded by the software and transferred to a standardized data collection sheet. In addition to performance variables, demographic data were collected, including age, sex, and dominant hand.

### 2.9. Statistical Analysis

Statistical analyses were performed using standard statistical software. Continuous variables were expressed as mean ± standard deviation (SD). Participants were grouped by age decades, and differences between groups were assessed using one-way analysis of variance (ANOVA). Normality of the data distribution was evaluated using the Shapiro–Wilk test. When significant differences were observed, post hoc pairwise comparisons were performed using Bonferroni correction. Effect sizes for the overall between-group comparisons were calculated using eta squared (η^2^), whereas effect sizes for pairwise comparisons between age groups were calculated using Hedges’ g to provide a standardized estimate of the magnitude of between-group differences. A *p*-value < 0.05 was considered statistically significant.

In addition, multiple linear regression analyses were performed to assess the association between age and DTC outcomes after adjustment for sex. Motor DTC, Cognitive DTC, and Mean DTC were separately entered as dependent variables, with age as a continuous independent variable and sex as a covariate. Unstandardized regression coefficients (B), 95% confidence intervals (95% CI), standardized coefficients (β), *p*-values, and adjusted R^2^ values were reported.

To determine whether sex modified the association between age and DTC, additional regression models including an age × sex interaction term were performed for Motor DTC, Cognitive DTC, and Mean DTC. Age was mean-centered before constructing the interaction term to facilitate interpretation of the model coefficients.

## 3. Results

### 3.1. Participant Characteristics and Performance

A total of 400 healthy participants were included in the study. For descriptive age-stratified analyses, participants were grouped into seven age categories: 18–29, 30–39, 40–49, 50–59, 60–69, 70–79, and ≥80 years.

As shown in Table 1, both motor and cognitive performance declined progressively with increasing age in both single-task (ST) and dual-task (DT) conditions. In the motor domain, baseline performance remained relatively stable between the third and fourth decades, followed by a gradual decline from the fifth decade onwards, with a more pronounced reduction after the age of 60. A similar pattern was observed for cognitive performance, although the decline appeared more evident under dual-task conditions.

Dual-task cost increased consistently across age decades for both motor and cognitive components, with a marked acceleration from the sixth decade onwards. Cognitive dual-task cost increased across older age groups, with larger differences becoming evident in later decades. Consequently, mean dual-task cost showed a progressive rise across decades, reaching the highest values in participants aged 80 years and older.

Statistical analysis confirmed significant differences across age decades for all variables (*p* < 0.001). Effect size analysis revealed large effects for motor performance (η^2^ = 0.63 for ST and 0.53 for DT), as well as for mean dual-task cost (η^2^ = 0.50), indicating a strong influence of age on dual-task performance. Moderate-to-large effect sizes were also observed for cognitive measures and dual-task cost components, further supporting the robustness of the age-related decline.

### 3.2. Motor and Cognitive Performance Across Age

**Figure 2:**
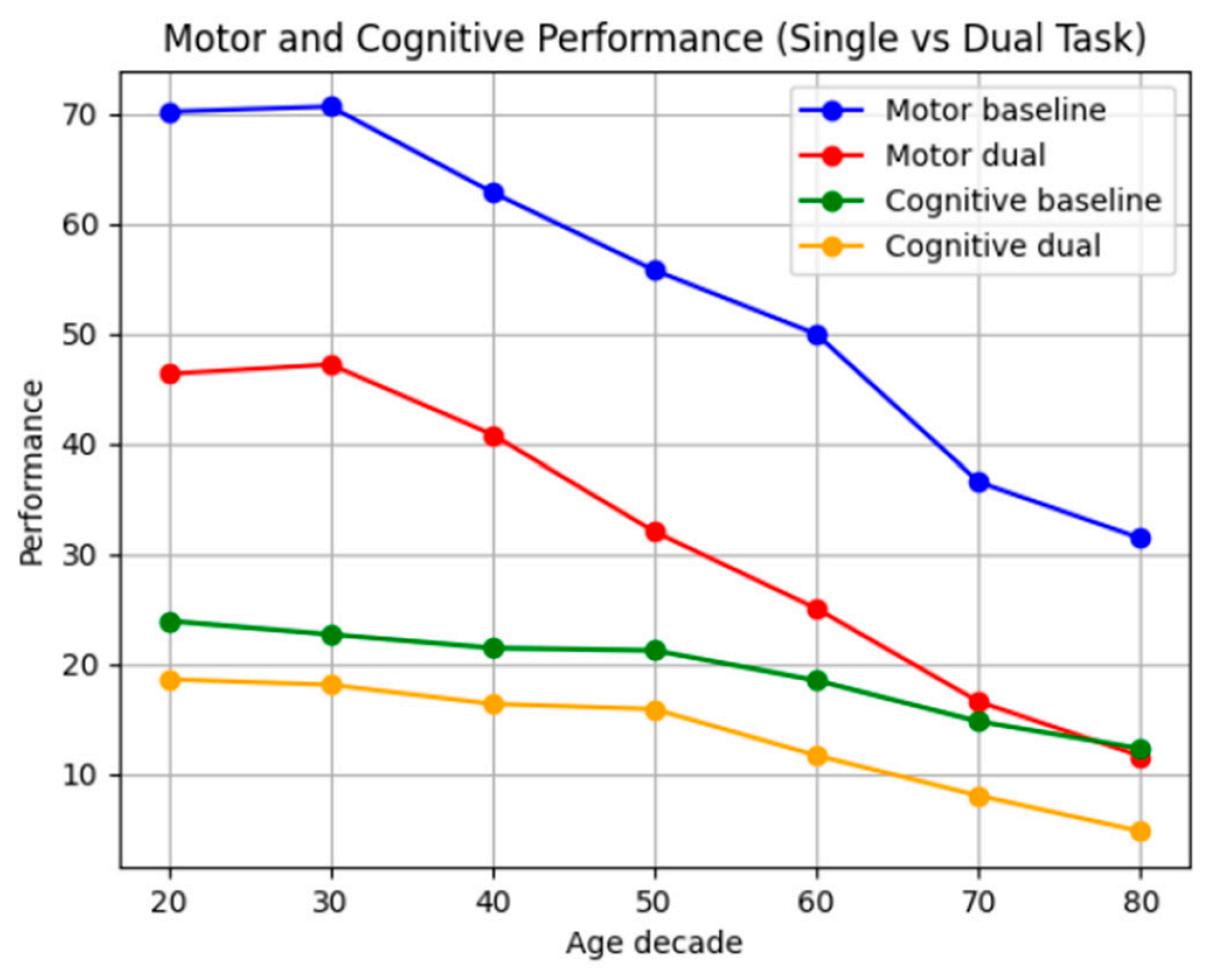
**Age-related changes in motor and cognitive performance under single-task and dual-task conditions.** Mean motor and cognitive performance during single-task (ST) and dual-task (DT) conditions across age decades. Motor performance was quantified as the number of successful target hits, whereas cognitive performance was expressed as the net cognitive score. Both domains showed a progressive age-related decline, which was more pronounced under dual-task conditions, indicating increasing cognitive-motor interference with aging. Values represent group means.

Motor and cognitive performance in both single-task (baseline) and dual-task conditions across age decades are shown in Figure 2.

#### 3.2.1. Motor Performance

Motor performance remained relatively stable across early and middle adulthood under single-task conditions. A gradual decline was observed with increasing age, particularly from the sixth decade onward.

Under dual-task conditions, motor performance was consistently lower than in the single-task condition across all age groups. This difference became more pronounced with advancing age, indicating increased susceptibility of motor function to cognitive interference in older individuals.

#### 3.2.2. Cognitive Performance

Cognitive performance showed a progressive decline with age in both single-task and dual-task conditions.

In younger participants, the difference between single-task and dual-task cognitive performance was relatively small. However, with increasing age, particularly after midlife, cognitive performance under dual-task conditions declined more markedly than in single-task conditions.

#### 3.2.3. Interaction Between Motor and Cognitive Domains

Across all age groups, performance in dual-task conditions was lower than in single-task conditions for both motor and cognitive domains.

Importantly, the gap between single-task and dual-task performance widened progressively with age, reflecting an increasing difficulty in managing simultaneous motor and cognitive demands.

### 3.3. Dual-Task Cost Differences Between Decades

**Figure 3:**
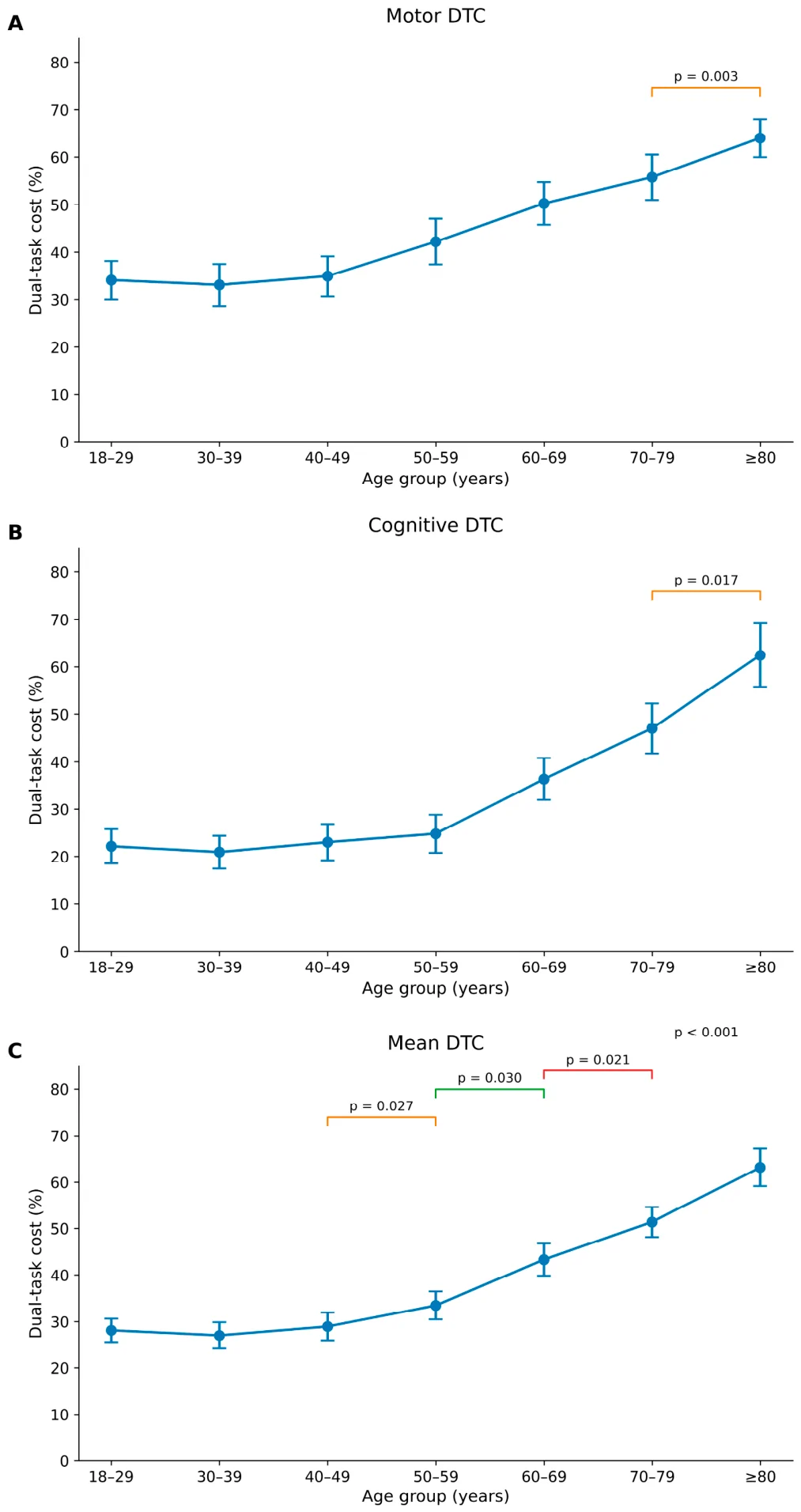
**Age-stratified dual-task cost and pairwise comparisons between consecutive age groups.** Mean Motor DTC (**A**), Cognitive DTC (**B**), and Mean DTC (**C**) are presented with 95% confidence intervals across age groups. Horizontal brackets indicate statistically significant pairwise comparisons between consecutive age groups based on Bonferroni-adjusted post hoc tests. Exact adjusted *p*-values are shown for significant comparisons. Non-significant comparisons are not displayed to improve readability.

Pairwise comparisons between consecutive decades are illustrated in Figure 3, with statistical significance indicated based on Bonferroni-adjusted post hoc comparisons (*p* < 0.05).

#### 3.3.1. Motor DTC

Motor dual-task cost showed progressively higher values across older age groups. Differences between consecutive decades were generally small or moderate and were not consistently statistically significant, suggesting a relatively gradual pattern of age-associated differences in motor interference.

#### 3.3.2. Cognitive DTC

Cognitive dual-task cost showed a clearer age-associated pattern. Differences between younger age groups were generally small and not statistically significant, whereas larger and statistically significant differences emerged in comparisons involving older age groups. This pattern suggests that cognitive interference under dual-task conditions is more pronounced at older ages.

#### 3.3.3. Mean DTC

Mean dual-task cost showed the most consistent age-associated pattern, with relatively stable values across younger age groups and progressively larger differences in comparisons involving older participants.

Effect-size analyses complemented the Bonferroni-adjusted pairwise comparisons, showing that the magnitude of between-group differences generally increased for contrasts involving older age groups. This pattern was particularly evident for Mean DTC, with large standardized differences observed between 50–59 and 70–79 years (Hedges’ g = 1.50), 50–59 and ≥80 years (g = 2.56), 60–69 and ≥80 years (g = 1.47), and 70–79 and ≥80 years (g = 0.96). Complete Bonferroni-adjusted pairwise comparisons and corresponding Hedges’ g effect sizes for Motor DTC, Cognitive DTC, and Mean DTC are presented in Table 2.

To facilitate interpretation at the individual level, age-stratified percentile values were calculated for Motor DTC, Cognitive DTC, and Mean DTC (Table 3). The distribution of Mean DTC shifted progressively toward higher values across older age groups, with the median increasing from 27.47 in participants aged 18–29 years to 61.89 in those aged ≥80 years. Similar age-associated patterns were observed for Motor and Cognitive DTC. These percentiles provide descriptive age-stratified reference values and should not be interpreted as diagnostic thresholds.

Multiple linear regression analyses confirmed that age remained significantly associated with DTC after adjustment for sex. Each additional year of age was associated with an increase of 0.506 points in Motor DTC (95% CI 0.422–0.589; standardized β = 0.513; *p* < 0.001), 0.582 points in Cognitive DTC (95% CI 0.497–0.667; standardized β = 0.557; *p* < 0.001), and 0.544 points in Mean DTC (95% CI 0.483–0.604; standardized β = 0.659; *p* < 0.001). The corresponding adjusted R^2^ values were 0.265, 0.319, and 0.445, respectively. Sex was also independently associated with DTC, with males showing lower adjusted Motor DTC (B = −4.19, 95% CI −7.74 to −0.65; *p* = 0.021), Cognitive DTC (B = −5.96, 95% CI −9.58 to −2.34; *p* = 0.001), and Mean DTC (B = −5.07, 95% CI −7.65 to −2.50; *p* < 0.001).

### 3.4. Sex Differences and Age × Sex Interaction

In the unadjusted comparison, no significant overall difference in dual-task cost was observed between males and females (*p* = 0.365). However, after adjustment for age in the multivariable regression analyses, males showed significantly lower Motor DTC (B = −4.19, 95% CI −7.74 to −0.65; *p* = 0.021), Cognitive DTC (B = −5.96, 95% CI −9.58 to −2.34; *p* = 0.001), and Mean DTC (B = −5.07, 95% CI −7.65 to −2.50; *p* < 0.001) than females.

**Figure 4:**
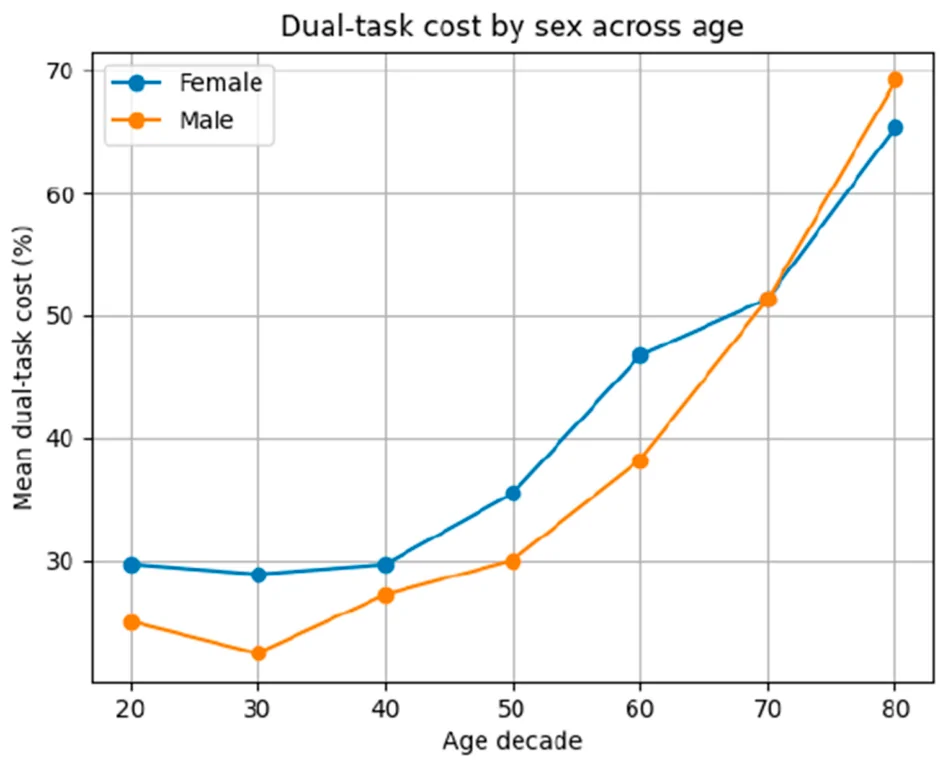
**Sex differences in dual-task cost across age decades.** Mean dual-task cost (DTC) in males and females across age decades. Mean DTC increased across older age groups in both sexes. Although age-adjusted analyses showed lower Mean DTC values in males, the age × sex interaction was not statistically significant (*p* = 0.649), indicating no evidence that sex modifies the association between age and Mean DTC. Error bars represent standard deviation.

Mean DTC values across age decades for males and females are shown in Figure 4. Both sexes exhibited higher Mean DTC values across older age groups, with broadly similar age-associated patterns.

To formally assess whether sex modified the association between age and dual-task cost, age × sex interaction terms were tested. No significant interaction was observed for Motor DTC (B = 0.127, 95% CI −0.047 to 0.302; *p* = 0.152), Cognitive DTC (B = −0.069, 95% CI −0.247 to 0.110; *p* = 0.450), or Mean DTC (B = 0.029, 95% CI −0.098 to 0.157; *p* = 0.649). Thus, although adjusted DTC levels differed between sexes, there was no evidence that sex significantly modified the association between age and DTC in this cohort.

## 4. Discussion

The present study provides a comprehensive analysis of dual-task performance across the adult lifespan, showing a progressive decline in both motor and cognitive domains, which becomes more pronounced under dual-task conditions.

Our findings demonstrate that dual-task performance deteriorates with age not only due to a reduction in baseline motor and cognitive capacities, but also due to an increased difficulty in managing simultaneous task demands. Older age was associated with lower baseline motor and cognitive performance and with greater difficulty managing concurrent task demands, with larger differences observed under dual-task conditions. This is consistent with previous literature indicating that dual-task paradigms are particularly sensitive to age-related functional decline [14].

Importantly, our results showed marked age-associated differences in cognitive performance under dual-task conditions. This pattern indicates a substantial cognitive contribution to the overall cognitive-motor interference observed across age groups and is consistent with the concept of limited attentional resources and competition between cognitive and motor processes [5,6].

This cross-sectional pattern is consistent with lower cognitive-motor integration efficiency at older ages. From a neurophysiological perspective, this decline may be explained by age-related structural and functional changes in key brain regions involved in dual-task processing, including the prefrontal cortex, basal ganglia, and cerebellum, as well as reduced white matter integrity [4]. These changes may impair the allocation of attentional resources and reduce the ability to process multiple streams of information simultaneously.

Furthermore, the observed association between older age and higher dual-task cost supports the potential value of dual-task paradigms for assessing cognitive-motor interference. However, most previous evidence regarding functional decline and fall risk has been derived from gait-based dual-task paradigms. Although gait-based and computer-based paradigms share the principle of competing cognitive and motor demands, they should not be considered physiologically equivalent. Gait additionally requires postural control, balance, locomotor coordination, and continuous sensorimotor integration, whereas the motor component of the present paradigm predominantly involves visuomotor coordination and upper-limb motor control. Therefore, findings from gait-based studies provide a useful conceptual framework for cognitive-motor interference but should not be directly extrapolated to the digital paradigm used in the present study [2,15].

An additional aspect to consider is the variability in task prioritization strategies observed during test performance. Some participants appeared to prioritize the cognitive task, maintaining higher accuracy at the expense of motor performance, whereas others prioritized the motor task, preserving motor output while reducing cognitive accuracy. This phenomenon, commonly described as task prioritization or “posture-first” or “cognition-first” strategies, has been consistently reported in dual-task paradigms [6]. More recent evidence suggests that individuals dynamically reallocate attentional resources depending on task demands and perceived risk, particularly in aging populations [16,17]. Such variability reflects the flexible but limited nature of cognitive resource allocation under dual-task conditions. In this context, Mean DTC was explored as a composite measure integrating motor and cognitive interference and may provide a global estimate that is less dependent on whether an individual preferentially preserves motor or cognitive performance. However, this potential advantage should be balanced against an important limitation: averaging both components may obscure domain-specific patterns, as similar Mean DTC values may result from substantially different combinations of motor and cognitive costs. Therefore, Mean DTC should be interpreted alongside motor and cognitive DTC rather than as a replacement for these domain-specific measures. The present study was not designed to establish the superiority of Mean DTC over its individual components, and further validation is required to determine its psychometric and clinical utility.

The more pronounced age-associated differences observed in Cognitive DTC and Mean DTC suggest that these measures may warrant further investigation as potentially sensitive indicators of cognitive-motor interference across the adult lifespan. However, the present findings do not establish Cognitive DTC or Mean DTC as clinical biomarkers. In particular, this study cannot determine diagnostic cut-offs or establish their ability to discriminate physiological age-associated changes from pathological cognitive decline. Validation in independent clinical cohorts, including individuals with mild cognitive impairment and neurodegenerative disorders, will be required to determine diagnostic accuracy, sensitivity, specificity, and clinically meaningful threshold values.

Sex-related findings were more nuanced after adjustment for age. Although the unadjusted comparison did not identify a significant overall difference between males and females, multivariable analyses showed lower adjusted DTC values in males. However, no significant age × sex interaction was identified for Motor, Cognitive, or Mean DTC, providing no evidence that sex modifies the association between age and dual-task interference in this cohort. These findings suggest that sex may influence overall DTC levels while the age-associated pattern remains broadly comparable between males and females.

A key finding of this study is that the increase in dual-task cost becomes more evident from approximately the fifth decade of life. This is consistent with evidence indicating that subtle cognitive decline and reduced processing efficiency may begin in midlife, even in healthy individuals [18,19]. These findings support the notion that brain aging is a gradual process that starts earlier than traditionally assumed.

The observation that age-associated differences in DTC become more evident from midlife may be relevant for future studies examining whether cognitive-motor assessments can contribute to the characterization of brain health trajectories before older age. Longitudinal and interventional studies will be required to determine the clinical significance of these cross-sectional findings and whether changes in dual-task performance are modifiable through targeted interventions. Increasing evidence supports the idea that interventions targeting cognitive function, physical activity, and combined cognitive-motor training may contribute to preserving cognitive and brain health across adulthood [20,21].

In this context, interventions aimed at maintaining cognitive-motor integration, such as dual-task training, may play a key role [22]. This approach is aligned with the concept of brain health promotion and proactive strategies across the lifespan. Recent studies have shown that targeted interventions can improve outcomes in populations with mild cognitive impairment [23], reinforcing the relevance of early intervention.

Importantly, the present findings support the feasibility of digital technologies for the objective and scalable assessment of cognitive-motor dual-task performance across adulthood. However, the present study did not include independent measures of everyday functioning, and therefore performance on the Dualtask Test should not be interpreted as a direct measure of broader functional capacity. In this context, the Dualtask Test represents an accessible digital assessment, as it is available online and free for use, enabling its evaluation in future research and clinical studies.

A key methodological feature of the present approach is that each participant acts as their own reference. By comparing single-task (motor and cognitive) performance with dual-task performance within the same individual, the influence of baseline inter-individual differences on the calculation of DTC may be reduced, allowing a more individualized estimation of cognitive-motor interference. However, this within-subject approach does not eliminate between-subject variability or potential confounding when DTC values are compared across age groups.

The availability of age-stratified reference data across the adult lifespan provides a framework for future studies in neurological populations. Establishing reference values enables future studies in neurological populations, such as Parkinson’s disease or cognitive impairment, and may also allow longitudinal monitoring to differentiate normal aging from pathological decline at the individual level.

One of the strengths of this study is the use of a standardized digital assessment tool in a relatively large sample covering a wide age range. In addition, the same assessment protocol was applied across recruitment settings in Spain and Ecuador.

An important aspect of the present study is the use of a relatively simple and accessible dual-task paradigm to capture meaningful information about cognitive-motor integration across the adult lifespan. In recent years, there has been growing recognition that well-designed, low-complexity assessment tools can provide valuable insights into complex physiological and cognitive processes, particularly when combined with digital platforms that enable standardized data collection and scalability. In this context, the present findings support the notion that simple, reproducible tests may contribute to advancing the understanding of age-associated differences in cognitive-motor interference, while also facilitating broader data acquisition and potential clinical translation. Such approaches may help bridge the gap between experimental research and real-world application, particularly in the field of digital health and neurorehabilitation.

However, several limitations should be considered. First, participants were recruited using a convenience sampling strategy, which may have introduced selection bias, as individuals recruited through the research teams’ networks and clinical settings may not fully represent the general population. Because the study did not use a population-based sampling frame and information on eligible non-participants was not systematically collected, the magnitude and direction of this potential selection bias could not be quantitatively assessed. Future studies should therefore use population-based or probabilistic sampling strategies to confirm the generalizability of the present age-stratified findings.

Second, no formal a priori sample-size calculation was performed because of the exploratory nature of the study, and sample sizes were unequal across age strata, with fewer participants represented in the oldest age groups. Although the overall sample of 400 participants allowed characterization of age-associated patterns across a wide age range, estimates derived from smaller strata may be less precise. Future studies should prospectively define age-stratified sample sizes and ensure adequate representation of the oldest age groups to improve the precision and robustness of age-specific reference estimates.

Third, several potentially relevant confounding variables were not systematically collected. These included educational level, physical fitness, habitual physical activity, socioeconomic status and previous computer use and mouse proficiency. Although DTC is calculated within subjects by comparing dual-task performance with each participant’s own single-task performance, this approach does not eliminate potential confounding in comparisons between age groups. Similarly, although all participants completed a standardized training phase before testing, this cannot fully eliminate differences in pre-existing digital familiarity. In addition, hardware characteristics, including screen size and resolution, mouse model, and mouse sensitivity, were not standardized across testing sites. However, single-task and dual-task conditions were performed during the same session using the same computer, screen, mouse, and hardware configuration for each participant, thereby reducing the potential influence of hardware variability on the within-subject DTC calculation. Nevertheless, an effect on absolute performance or between-participant variability cannot be excluded. Future studies should prospectively collect relevant participant-level variables and incorporate them into multivariable models, while also systematically recording and, where feasible, standardizing hardware characteristics or directly assessing the robustness of DTC across different computer configurations. Geographical and sociocultural variables should also be recorded at the individual level to allow their potential influence to be formally evaluated.

Fourth, standardized cognitive screening with the MoCA was additionally performed only in participants aged over 70 years as a precautionary measure to reduce the likelihood of including older individuals with unrecognized cognitive impairment. Therefore, subtle or subclinical cognitive impairment cannot be completely excluded in younger and middle-aged participants. Future studies should apply standardized cognitive screening across the entire age range to minimize this potential source of residual confounding.

Fifth, the motor component of the present paradigm is a computer-based upper-limb visuomotor task and differs substantially from gait-based dual-task paradigms commonly used in the literature. Consequently, the present findings should not be directly extrapolated to gait performance, postural control, fall risk, or real-world mobility. Future studies should directly compare digital and gait-based dual-task paradigms to determine the extent to which they capture shared versus task-specific components of cognitive-motor interference.

Sixth, the Dualtask Test has not yet undergone comprehensive psychometric validation. Although preliminary pilot testing was conducted to optimize task difficulty and duration, this was not designed to assess reliability or validity. Formal evaluation of test–retest reliability, construct and criterion validity, measurement error, and responsiveness is therefore required. Until such validation is completed, the age-stratified values reported in this study should be regarded as reference data rather than definitive normative values for individual clinical decision-making. Future studies specifically designed to establish the psychometric properties of the Dualtask Test, ideally including comparison with established cognitive-motor assessment paradigms, are therefore warranted. The absence of a universally accepted gold standard for dual-task assessment should also be considered when designing such validation studies [6].

Seventh, the cross-sectional design does not allow causal inference or assessment of intra-individual change over time. Therefore, the differences observed across age groups should be interpreted as age-associated cross-sectional patterns rather than evidence of longitudinal deterioration attributable to aging. Longitudinal cohort studies with repeated Dualtask Test assessments are required to characterize individual trajectories of change and determine the temporal evolution of DTC across the adult lifespan.

Finally, the present study was not designed to establish diagnostic thresholds or to evaluate the ability of DTC measures to distinguish physiological age-associated differences from pathological cognitive decline. Accordingly, the age-stratified values and percentiles reported here should be interpreted as descriptive reference data rather than clinical cut-offs. Future studies should include both healthy individuals and well-characterized clinical populations, including individuals with mild cognitive impairment and neurodegenerative disorders, to establish diagnostic accuracy, sensitivity, specificity, and clinically meaningful thresholds using appropriate methods such as receiver operating characteristic analyses.

## 5. Conclusions

This study provides age-stratified reference data for digital motor-cognitive dual-task performance across the adult lifespan using a standardized assessment protocol. Dual-task performance was lower and dual-task cost higher across older age groups, with particularly pronounced age-associated differences in Cognitive and Mean DTC. These findings characterize performance on this specific digital cognitive-motor paradigm and provide a reference framework for its further investigation. However, the present results do not establish the Dualtask Test as a measure of broader functional capacity or as a validated clinical biomarker. Further studies should establish its psychometric properties, evaluate its relationship with independent measures of everyday functioning, and determine its ability to distinguish physiological age-associated differences from pathological cognitive decline in well-characterized clinical populations.

Future longitudinal studies should determine the ability of these age-stratified reference to distinguish healthy aging from early pathological cognitive decline and to monitor disease progression and treatment response in neurological disorders.

## Figures and Tables

**Table 1:** Participant characteristics and dual-task performance across age decades. Demographic characteristics and motor and cognitive performance under single-task (ST) and dual-task (DT) conditions according to age decade. Data are presented as mean ± standard deviation. Dual-task cost (DTC) was calculated separately for motor and cognitive performance, and mean DTC was calculated as the arithmetic mean of both components. Between-group differences were assessed using one-way ANOVA, with effect sizes expressed as eta squared (η^2^). Abbreviations: ST, single-task; DT, dual-task; DTC, dual-task cost; M, males; F, females; η^2^, eta squared.

Age Decade	*N* (M/F)	Motor ST	Motor DT	Cognitive ST	Cognitive DT	Motor DTC (%)	Cognitive DTC (%)	Mean DTC (%)
18–29	79 (26/53)	70.44 ± 9.32	46.85 ± 10.51	24.20 ± 3.89	18.70 ± 4.73	34.03 ± 11.85	22.12 ± 13.39	28.08 ± 11.44
30–39	55 (16/39)	70.65 ± 9.17	47.20 ± 10.77	22.71 ± 4.13	18.13 ± 4.46	33.03 ± 11.24	20.90 ± 11.80	26.97 ± 10.62
40–49	48 (14/34)	62.85 ± 10.28	40.83 ± 10.79	21.48 ± 4.10	16.40 ± 4.72	34.86 ± 10.62	22.98 ± 11.92	28.92 ± 10.52
50–59	56 (20/36)	55.77 ± 10.60	32.05 ± 10.71	21.25 ± 4.04	15.91 ± 4.64	42.22 ± 11.74	24.76 ± 12.20	33.49 ± 11.21
60–69	67 (27/40)	49.96 ± 11.15	25.06 ± 10.35	18.52 ± 4.34	11.70 ± 4.67	50.25 ± 13.85	36.36 ± 14.51	43.31 ± 14.22
70–79	59 (18/41)	36.56 ± 10.56	16.59 ± 7.99	14.80 ± 4.21	8.05 ± 3.95	55.74 ± 11.88	46.98 ± 13.56	51.36 ± 12.35
≥80	36 (14/22)	31.44 ± 9.60	11.64 ± 5.85	12.47 ± 3.85	4.97 ± 3.27	63.96 ± 10.54	62.44 ± 13.41	63.20 ± 11.94
*p*-value		<0.001	<0.001	<0.001	<0.001	<0.001	<0.001	<0.001
η^2^		0.63	0.53	0.30	0.43	0.28	0.39	0.50

**Table 2:** Bonferroni-adjusted pairwise comparisons and Hedges’ g effect sizes for DTC outcomes: *p* values are Bonferroni-adjusted for the 21 pairwise age-group contrasts within each DTC outcome. Hedges’ g is signed so that positive values indicate higher DTC in the older age group. Absolute g values of approximately 0.2, 0.5, and 0.8 are commonly interpreted as small, moderate, and large effects, respectively.

Age-Group Comparison	Motor DTC *p* Adjusted	Motor DTC Hedges’ g	Cognitive DTC *p* Adjusted	Cognitive DTC Hedges’ g	Mean DTC *p* Adjusted	Mean DTC Hedges’ g
18–29 vs. 30–39	1.000	−0.057	1.000	0.122	1.000	0.041
18–29 vs. 40–49	1.000	0.049	1.000	0.172	1.000	0.133
18–29 vs. 50–59	0.223	0.450	0.002	0.684	0.001	0.679
18–29 vs. 60–69	<0.001	0.889	<0.001	1.275	<0.001	1.375
18–29 vs. 70–79	<0.001	1.189	<0.001	1.705	<0.001	1.872
18–29 vs. ≥80	<0.001	2.003	<0.001	2.215	<0.001	2.755
30–39 vs. 40–49	1.000	0.105	1.000	0.045	1.000	0.095
30–39 vs. 50–59	0.080	0.507	0.014	0.575	0.003	0.638
30–39 vs. 60–69	<0.001	0.947	<0.001	1.150	<0.001	1.331
30–39 vs. 70–79	<0.001	1.252	<0.001	1.571	<0.001	1.849
30–39 vs. ≥80	<0.001	2.075	<0.001	2.098	<0.001	2.756
40–49 vs. 50–59	0.547	0.399	0.056	0.524	0.027	0.540
40–49 vs. 60–69	<0.001	0.840	<0.001	1.102	<0.001	1.236
40–49 vs. 70–79	<0.001	1.140	<0.001	1.526	<0.001	1.740
40–49 vs. ≥80	<0.001	1.931	<0.001	2.054	<0.001	2.637
50–59 vs. 60–69	0.238	0.431	0.169	0.481	0.030	0.520
50–59 vs. 70–79	<0.001	0.744	<0.001	0.958	<0.001	1.503
50–59 vs. ≥80	<0.001	1.545	<0.001	1.486	<0.001	2.562
60–69 vs. 70–79	1.000	0.305	0.318	0.468	0.021	0.599
60–69 vs. ≥80	<0.001	1.103	<0.001	1.012	<0.001	1.466
70–79 vs. ≥80	0.003	0.797	0.017	0.555	<0.001	0.963

**Table 3:** Age-stratified percentile reference values for Motor, Cognitive, and Mean dual-task cost (DTC): Values represent the 25th (P25), 50th (P50; median), and 75th (P75) percentiles of the observed DTC distribution within each age group. These percentiles are intended as age-stratified reference values and should not be interpreted as diagnostic cutoffs.

Age Group	*n*	Motor DTC P25	P50	P75	Cognitive DTC P25	P50	P75	Mean DTC P25	P50	P75
18–29	79	20.26	31.03	48.75	11.32	17.86	31.22	20.45	27.47	35.38
30–39	55	21.58	31.03	41.50	10.44	18.18	29.10	21.04	28.26	32.10
40–49	48	23.68	33.63	43.42	14.16	22.22	31.33	20.97	29.50	36.10
50–59	56	28.88	42.18	57.89	13.98	22.90	33.82	25.31	34.02	39.24
60–69	67	36.32	50.00	62.38	24.26	32.14	50.00	32.43	42.20	52.72
70–79	59	44.38	57.14	70.33	33.33	46.67	60.00	43.46	51.25	60.63
≥80	36	55.21	64.52	75.00	50.00	58.33	77.69	56.70	61.89	70.68

## Data Availability

The datasets generated and analyzed during the current study are not publicly available because they contain information that could compromise participant privacy and are subject to ethical restrictions. Requests to access the data will be considered by the corresponding author on a case-by-case basis, subject to approval by the relevant Ethics Committee and compliance with institutional and data protection regulations.

## Abbreviations

The following abbreviations are used in this manuscript:

ST	Single task
DT	Dual Task
DTC	Dual Task Cost
